# Phase I non-randomized clinical trial of allogeneic natural killer cells infusion in acute myeloid leukemia patients

**DOI:** 10.1186/s12885-023-11610-x

**Published:** 2023-11-10

**Authors:** Mohammad Ahmadvand, Mahdieh Shokrollahi Barough, Maryam Barkhordar, Ali Faridfar, Afshin Ghaderi, Hasan Jalaeikhoo, Mohsen Rajaienejad, Keivan Majidzadeh, Ardeshir Ghavamzadeh, Ramin Sarrami-Forooshani

**Affiliations:** 1 Cell Therapy and Hematopoietic Stem Cell Transplantation Research Center, Research Institute for Oncology, Hematology and Cell Therapy, Tehran University of Medical Sciences, Tehran, Iran; 2 ATMP Department, Breast Cancer Research Center, Motamed Cancer Institute, ACECR, P.O. BOX: 15179/64311, Tehran, Iran; 3 Research Center for Cancer Epidemiology and Screening, Aja University of Medical Sciences, Tehran, Iran; 4 Department of Internal Medicine, Hematology and Medical Oncology Ward, Yasuj University of Medical Sciences, Yasuj, Iran; 5 Genetics Department, Breast Cancer Research Center, Motamed Cancer Institute, ACECR, Tehran, Iran; 6 Cancer and cell therapy research center, Tehran University of Medical Sciences, Tehran, Iran

**Keywords:** Acute Myeloid Leukemia (AML), Allogeneic NK cell therapy, Adoptive transfer, Cellular therapy

## Abstract

**Introduction:**

A new type of immune cell transplantation called allogeneic NK cell infusion is proposed as a potential universal off-the-shelf cell product for adoptive immune cell therapy in hematologic malignancies.

**Design:**

A multicentral phase I non-randomized clinical trial was conducted to assess the safety, feasibility, and potential efficacy of adoptively infused NK cells in patients with refractory/relapsed AML. We evaluated the feasibility of the trial by considering cell production, patient selection, and treatment protocol.

**Method:**

Allogeneic NK cells were produced from random healthy unrelated donors; 10 patients were selected according to the inclusion criteria and were included in two groups in case of NK cell dose escalation. Two cell infusions were given, spaced 7 days apart, following a lymphodepletion conditioning regimen of fludarabin-endoxan administered 7 days before the first infusion. The intervention safety was scored using Common Terminology Criteria for Adverse Events (CTCAE) based on variations in vital signs due to cell infusion. NK cell chimerism, tumor burden, and duration of relapse were considered to be components of efficacy. The pilot feasibility evaluation was checked using the CONSORT platform.

**Results:**

The NK cell infusion procedure was well tolerated, and no grade 2–5 toxicities related (possible or probable) to PB-NK cell infusion were observed. Four patients developed grade 1 transient chills, headaches, vomiting, and bone pain following each PB-NK cell infusion that were not required hospitalization. One of these patients (p01) died because of severe acute respiratory syndrome. Of 9 evaluable patients, 6 (66.6%) showed stable disease (SD) and 3 (33.3%) presented progressive disease (PD). Of 6 SD patients, 2 (p08 and p09) remained alive in SD and 3 patients (p04, p05 and p07) converted to PD at 9 months after infusion of NK cells, and 1 (p03) was not evaluable due to follow-up loss. No patient achieved complete remission.

**Conclusion:**

The study demonstrated the feasibility and safety of adoptive transfer of random healthy unrelated donor PB-NK cells in refractory/relapsed AML patients and supports continued study in phase II clinical trials in relapsed/refractory AML patients.

**Supplementary Information:**

The online version contains supplementary material available at 10.1186/s12885-023-11610-x.

## Introduction

AML is a hematologic malignancy that frequently affects elderly individuals, with a median diagnosis age of 68 years. However, the survival rate within a five-year timeframe is only 26%, which is concerning [1, 2]. Although survival rates for AML patients have not improved much in the last 30 years, certain chemotherapy treatments can lead to complete remission (CR) in younger patients. However, these remissions are not long-lasting, and consolidation treatment is necessary for a better long-term outcome [1]. In addition to conventional treatments, allogeneic hematopoietic stem cell transplantation (HSCT) is an efficient treatment for patients. This treatment acts by CD34 + healthy cell replacement in line with antileukemia immune response stimulation that is mediated by natural killer (NK) and alloreactive T cell responses. Due to the greater risk of treatment-related complications and death, many elderly patients with AML are ineligible for allo-HSCT. Innovative therapies using immune cells that offer graft-versus-leukemia (GVL) effects similar to those of NK or T cells show great potential as a means of providing cell-based treatments for AML without the need for allo-HSCT [3].

AML prognosis at a younger age is worst, and patient-related studies have shown that the disease is fatal in younger adults; however, bone marrow transplantation is well tolerated in young people, so advanced therapy with alloimmune cell transplantation might be very promising in this category [4]. Allogeneic NK cell transplantation as one of these advanced therapies is prescribed via intravenous injection of ex vivo expanded NK cells. The scientific background of NK cells indicates that they are one of the most valuable categories of intrinsic immunity against tumors, which have a significant capacity to lyse malignant cells both in vitro [5] and in vivo [6]. It has been shown that cytotoxic activity of NK cells is associated with a reduced frequency of cancer [7], suggesting that NK cells contribute to tumor immune surveillance [8]. In clinical trials, there is sufficient evidence that allogeneic NK cells can contribute to GVL effects in the context of allo-HSCT [9–11], paving the way for assessing NK cell-based approaches to treat hematologic malignancies [12].

In recent decades, numerous methods have been explored to utilize NK cells in therapeutic applications [13]. Published trials on adoptive allogeneic NK cell therapy in a transplant and non-transplant setting have displayed well-tolerated outcomes after NK cell infusion in line with PR/CR in some patients [14–19]. In order for NK cell graft improvement, a conditioning regimen should be used, in addition to the mild tumor burden decrement, it can cause bone marrow depletion and reinforce the NK cell grafting [16].

The most studied trial for NK cell transplantation is for haploidentical sources, and the safety and efficacy of allogeneic NK cell infusion were confirmed in patients with hematologic malignancy from haploidentical donors. The haploidentical selection was performed to avoid NK cell transplant rejection and potential immune stimulation. However, studies have proven that allogeneic cells are also well tolerated in the recipient’s body, which is due to killer-cell immunoglobulin-like receptors (KIRs) [16]. (KIR/KIR-ligand mismatch), can recognize and react to this missing self and mediate cytotoxicity, so has a crucial impact on alloreactivity, which is favorable to tumor cell elimination [20]. There is poor evidence regarding non-related allogeneic NK cell transplantation in malignancy disorders, especially in AML. Our previous study on non-related allogeneic NK cell infusion in acute SARS-COV2 infection with low respiratory symptoms was a safety and feasibility evaluation in a pilot phase 1 non-randomized clinical trial. The results of the study showed no adverse effect due to the intervention [21]. Although that project had no conditioning regimen and the aim of the study was to investigate the antiviral adjuvant function of NK cell therapy, we became passionate about focusing on the adverse effect evaluation of non-related allogeneic NK cell infusion in AML patients with bone marrow depleting regimen. However, this is the first report of ex vivo-expanded allogenic NK cells derived from healthy unrelated donors in Iranian patients with refractory/relapsed AML. Therefore, on the basis of the GVL effects of peripheral blood NK (PB-NK) cells, we performed an efficient method for the large-scale ex vivo expansion of NK cells from peripheral blood mononuclear cells (PBMC) from random healthy unrelated donors under good manufacturing practice (GMP) conditions [22, 23].

Here, we report the results of a phase I adoptive cancer immune cell therapy with two infusions from ex vivo expanded random unrelated healthy donors PB-NK cells administered to patients with refractory/relapsed AML, which can result in some cells having a complete donor–recipient KIR ligand mismatch. This is a pilot study for safety and dose escalation analysis that focuses on the feasibility of production, administration, performance, safety, and follow-up for short-period efficacy analysis.

We deliberately focused on the KIR mismatch in this pilot project to obtain evidence of the association of this characteristic with the intervention outcome. This approach not only permits the extended feasibility of MHC class I mismatch but also overcomes the limitations of small potential donor pools.

The primary objectives were to determine the safety and feasibility of this adoptive cell therapy in patients with pre-conditioned AML, and the secondary objective was to evaluate the probable antitumor efficacy of PB-NK cells. This study should be executed as a non-randomized trial because it is a controlled study to evaluate the feasibility of production, implementation, safety, and access to patient follow-up, and blinding or randomization might complicate the evaluation of the main objective. One significant drawback of this study is its small sample size. It only provides sufficient data to assess feasibility. To obtain more accurate and comprehensive results, a phase 2 study with a one-year follow-up is necessary. However, the publication of these findings will take time, and we are passionate about reporting these preliminary data until phase II report completion.

## Materials and methods

### Study Design

This is a non-parallel, pilot clinical trial that was performed as a non-randomized, non-blinded study. It is a multi-central trial that has been registered in the Iranian clinical trial data set (IRCT) coded IRCT20200621047859N3 on 30/12/2020 and achieved the ethics code before patient enrollment (IR.ACECR.IBCRC.REC.1398.015) 16/10/2019. There was no preference in the selection of patients for dose administration, and patient enrollment was done in chronological order and was performed on a timeline from the beginning of the project.

This study was executed by Motamed Cancer Institute and Cell Therapy and Hematopoietic Stem Cell Transplantation Research Center of Shariati Hospital of Tehran University of Medical Sciences. Cell infusion was administered in the Department of Oncology of Imam Reza Hospital. NK cell chimerism was performed on days 2, 7, and 28 after infusion, as previously described [17]. Chimerism investigations of immunologically sorted NK cells were performed using a standard variable number of tandem repeat methods [24].

Due to the dose escalation evaluation, all patients were enrolled in cohorts of 5 per dose level. Both groups were selected based on the same inclusion criteria and treated with a unique conditioning regimen, and the PB-NK-cell product was administered at days 0 and + 7. Cohort 1 was initiated with an adoptive transfer dose of 2 × 10^6^ cells/kg for the first injection and 5 × 10^6^ cells/kg for the second infusion (once weekly, two infusions). Cohort 2: Patients received 5 × 10^6^ cells/kg for the first infusion and 10 × 10^6^ cells/kg for the second infusion, once weekly, two infusions). The Cohort 1 intervention was performed earlier than that of Cohort 2, and the Cohort 2 study was started after the safety assessment completion of Cohort 1.

### Feasibility evaluation criteria

Due to the CONSORT guidelines, the major evaluated parameters for the pilot study were considered follows [25].

- **Sample size evaluation**: 5 patients’ enrollment in each cohort was considered minimal statistical patients that can be evaluated in case of safety and follow-up accessibility. The availability of patient files and regular monitoring is another parameter for study feasibility evaluation. Because finding the eligible patient to replace the excluded patients does not have a certain period of time, the minimum number of available patients is considered in determining the feasibility of the study, which is one of the criteria to ensure the feasibility of the sample size. We used the previous checklist that was filled out in the COVID-19 pilot study. For this evaluation, the categorical scoring was allocated according to the entered and excluded number of patients into special care units; 1, every two days admission; 2, every day admission; 3, every day two patients’ admission (Supplementary file 1).
- **Patient-related data accessibility** is considered another important criterion to evaluate feasibility, and it was graded as follows: 1, demographic information of a patient; 2, the patient’s hardcopy file; 3, local access to the patient’s digital file; 4, Web-based full access to the patient’s information for long-term follow-up; 5, full access to patient information. (Supplementary file 1)
- **Death cause evaluation** data in the hospital or personal physician report was graded from 0 to 2 for the exanimate patients: 0, no access to cause of death; 1, general information of death causes that are included in the patient’s file; 2, professional death causes declared by the evaluation of medical doctorate, which was associated with the trial. Both cohorts were evaluated for clinical symptoms using patient-related samples. A completed questionnaire was used for all assessments, and the main liaison researcher conducted them. The questionnaire was reviewed by the Ethics Committee (Supplementary file 1).
- **Cell production**: The feasibility of cell production was evaluated using production-related quality control and NK cell immunophenotyping, characterizing, and functional assays using K562 co-culture.

### Safety Assessment

The Common Terminology Criteria for Adverse Events (CTCAE) Version 4.03 in blood and hematologic disease was considered for safety assessment analysis. The CTCAE parameters based on clinical symptoms and vital signs were listed as heart attack, changes in respiratory capacity, fever, rash, and anaphylactic shock symptoms that were checked using remote patient monitoring (RPM) devices during cell infusion until 48 h. The patient ’s vital signs, laboratory readings, and physical recovery progress were closely monitored, recorded in their medical files, and graded using the CTCEA checklist (Supplementary file 2).

### Donor selection and blood sampling

Allogeneic unrelated healthy donors were randomly chosen from healthy volunteers after signing the consent form. All volunteers have been proven to have a negative active infection of SARS-COV2 via the gold standard RT-PCR test. The HIV, HBV, HCV, and blood culture examinations were also performed for all donors, and all results were negative. Finally, 70 ml of whole blood was obtained from the donors by intravenous blood sampling using blood collection syringes and injectors in heparinized falcon tubes. The samples were immediately transferred to a cleanroom under cold conditions. Donor selection is based on whether donors have either maximum KIR incompatibility against recipients or a potent KIR B haplotype.

### Patients

Ten patients aged 18–70 years with refractory/relapsed AML were enrolled in this trial. All included patients had failed standard therapy and were not candidates for further induction chemotherapy or eligible for allo-HSCT because of inadequate disease control or chemorefractory (Table 1). The included patients were eligible with PS ≥ 2 [Performance Status (PS)] and [glomerular filtration rate (GFR)] GFR ≥ 60 or creatinine ≥ 2 mg/dl, ALT < 2.5XULN / Bid ≤ 2, O2Sat > 92% in room air, cardiac classification status (III > NHC) or [ejection fraction] EF > 50%. The primary endpoints were the safety and feasibility of adoptive transfer of allogeneic donor NK cells in patients with refractory/relapsed AML. Secondary endpoints included assessment of probable antitumor response. 5 patients (No time preference) were admitted to Imam Reza Hospital and 5 other patients were admitted to Yasouj Hospital. The signed consent forms were checked by the ethics committee after each intervention.

### Preparation of NK cell-enriched products

Peripheral blood mononuclear cells (PBMCs) were isolated from healthy unrelated donors, and PB-NK cells were expanded as described previously [21, 22]. Briefly, CD3^+^ T-cell–depleted PBMCs (using the Miltenyi CliniMACS system) were expanded at a seeding density of 2 × 10^5^ cells/mL in X-VIVO 10 medium with 5% AB serum, 2 × 10^6^ irradiated autologous PBMCs ((2,500 rad), 10 ng/mL anti-CD3 monoclonal antibody (OKT3; Orthoclon-USA), and 500 IU/mL of MACS GMP recombinant human IL-2 (Miltenyi Biotec-USA). OKT3 was supplemented just once at the beginning of the expansion to stimulate the T cell population in the irradiated feeder cells. NK cells were fed fresh medium with 500 IU/mL of IL2 every 2–3 days to maintain cellular concentration at 1–2 × 10^6^ until they were harvested on day 21.

### NK cell product characterization

After expansion on culture day 20, cells were harvested and PB-NK cell purity was evaluated by flow cytometry (CD3^−^/CD56^+^). K562 cells were obtained from the Iranian biological resource center (IBRC-Iran) and cultured in RPMI-1640 medium (Life Science-USA) supplemented with 10% FBS (Life Science-USA).

To evaluate the functional status of the PB-NK cells using intracellular flow cytometry [interferon (IFN)-γ], CD107a degranulation (all antibodies were purchased from Biolegend-USA), and lactate dehydrogenase (LDH) release assay (Sigma-Germany) against K562 (IBRC-Iran)) as described previously [26] as targets. The cells were then washed using the Sepax System (Biosafe, Eysins, Switzerland) and suspended in 100 ml PlasmaLyte supplemented with 0.5% human serum albumin (HSA). All NK cell products met release criteria including negative Gram staining, endotoxin assay < 5 EU/kg patient weight, mycoplasma contamination, and visual inspection negative for contamination and cell viability of ≥ 80%.

### Conditioning regimen

**Fig. 1:**
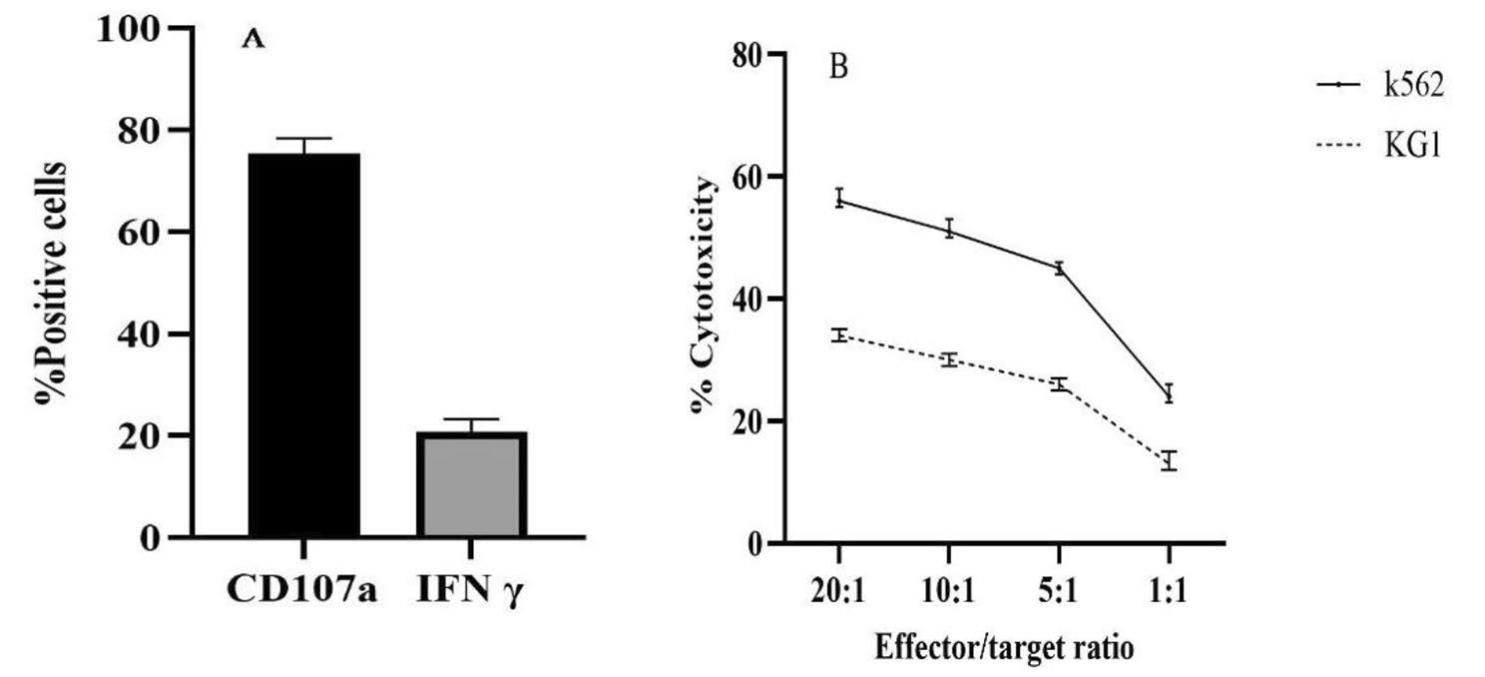
Expanded PB-NK cells reveal an active phenotype before infusion. **A**, Characterization of CD56^+^ cells in case of IFN-γ and CD107a expression against K562 targets (E:T ratio = 1:1). **B**, NK: K562 co-culture with the final product of NK cell expansion

The conditioning regimen was initiated at day 7 from NK-cell infusion and consisted of one infusion of fludarabine (f; 25 mg/m2) per day for the first 4 days (days − 7 to -4) followed by one infusion of cyclophosphamide (c; 25 mg/kg) per day (days-3 to-2). Fresh PB-NK cells were infused on day 0. The remaining PB-NK cells were cryopreserved (40% Plasmalyte, 50% human AB serum, 10% dimethyl sulfoxide) and thawed, washed, and infused on days + 7 (Fig. 1). After NK cell infusion, patients were followed weekly through month 1 and biweekly through month 3. To minimize unwanted side effects from intensive induction chemotherapy regimen in this heavily pretreated patient group with expected high treatment–related morbidity and possible mortality, we used a less toxic primary lymphodepletion regimen and omitted IL-2 subcutaneous administration.

### Patient monitoring and laboratory findings

The total white blood cell count (WBC) was reported for each patient before cell therapy (at the hospitalization date) and after cell therapy (1 week after cell therapy). The blast cell percentage was evaluated for each patient before cell therapy and 3 months after cell infusion.

The levels of alkaline phosphatase (ALP) with a normal range of 44 to 147 international units per liter (IU/L), aspartate aminotransferase (AST, SGOT) with a normal range of 12–38 U/L, alanine aminotransferase (ALT, SGPT) with a normal range of 53–120 µmol/L and potassium (K) with a normal range of 3.6–5.3 mEq/L, and sodium (Na) with a normal range of 40–220 mmol/day were evaluated every day, and the results before the first cell infusion and 48 h after cell injection were reported for each patient.

## Results

### Patients, Disease characteristics, and treatment schedule

10 patients with relapsed/refractory AML were included in this trial, and their disease characteristics are demonstrated in Table 1. 10 patients with refractory or relapsed AML enrolled in the study were considered ineligible for HSCT at the time of inclusion in the trial. The median age of the enrolled patients was 50.5 years (range, 29–61 years), and all patients had previously received multiple therapies for AML. All patients received lymphodepletion chemotherapy before the infusion of PB-NK cells. All recipients’ NK cells had transient engraftment of NK cells (median, 8 days; range, 2 to 28 days), with NK cell chimerism of 6% donor (range, 2–21%; Table 1). Four patients continued to have measurable donor NK cells on day 28 (range,8%to 21%).

**Table 1:** Patient demographics and baseline characteristics and engraftment patients feature

Patient	Age	Sex	% Blasts in the BM before infusion	WBC Count/mL	Hb (g/dL)	Platelet count	Recipient		Donor KIR Mismatch*	Peak NK ell Chimerism	
							HLA-Bw	HLA-C		Day	% Donor
P01	29	Female	65%	5050	10.5	65	6/6	C1/C1	2DL1, 3DL1	7	2
P02	51	Male	50%	800	9.3	27	4/6	C1/C2	NA	7	7
P03	37	Male	44%	2700	9.8	35	6/6	C1/C1	2DL1, 3DL1	2	5
P04	50	Male	21%	700	10.1	41	4/4	C1/C1	2DL1	7	9
P05	53	Male	30%	1800	8.6	39	4/6	C1/C1	2DL1, 3DL1	7	2
P06	61	Female	27%	1050	9.5	48	6/6	C1/C1	2DL1, 3DL1	28	8
P07	55	Mal	28%	1150	9.2	53	6/6	C1/C2	2DL2/3, 3DL1	7	4
P08	48	Female	35%	700	8.8	64	4/4	C2/C2	2DL2/3	28	21
P09	49	Male	28%	900	10.2	28	6/6	C1/C2	3DL1	28	11
P10	53	Female	26%	1700	10.6	36	4/6	C1/C1	2DL1	28	17

*Receptor-ligand model

### PB-NK cell product

PB-NK cell expansion to the target dose was reached in all 10 patients with a mean NK cell purity of 94.8% (91.3– 97.5%), and we were able to evaluate the cytotoxicity of these cells in some patients (5/10). In line with our preclinical data, the NK cells were highly functional with CD107a degranulation IFN-γ production in response to the K562 cell line (Fig. 1A) as well as in vitro cytotoxicity against K562 and AML cell lines (Fig. 1B).

### Safety and feasibility assessment

None of the 10 patients experienced dose-limiting toxicity (DLT) during PB-NK cell infusion or during the 28 days of the post-infusion observation period. One of these patients (P01) died 8 weeks after PB-NK cell infusion because of severe acute respiratory syndrome with a positive test for SARS-COV2 seven days after 2nd infusion. No grade 2–5 toxicities related (possible or probable) to PB-NK cell infusion occurred. Four patients developed grade 1 transient chills, headaches, vomiting and bone pain following each PB-NK cell infusion that were not required hospitalization (Table 2). In all cases, these impairments were reversible and responsive to supportive care, antipyretics, and intravenous hydration. On the basis of these data, we conclude that PB-NK cell infusion was safe and feasible, with 4 out of 10 patients having transient (< 6 h) and treatable adverse events (chill, headache, vomiting and bone pain) attributed to cell infusion.

**Table 2:** NCI Common Terminology Criteria for Adverse Events

NCI CTCAE term	Total, n	Grade 1, n	Grade 2, n	Grade 3, n	Grade 4, n	Grade 5, n
Chills	1	1	0	0	0	0
Nausea	0	0	0	0	0	0
Headache	1	1	0	0	0	0
Vomiting	1	1	0	0	0	0
Encephalitis infection	0	0	0	0	0	0
Sinus tachycardia	0	0	0	0	0	0
Bone pain	1	1	0	0	0	0
Pain in extremity	0	0	0	0	0	0
Rash maculopapular	0	0	0	0	0	0

Number and grade of AEs that were considered to be either probable or possibly related to treatment with infusion of allogenic PB-NK cells. AEs related to conditioning, including fludarabine and cyclophosphamide, are not reported in this table

The feasibility-related criteria were evaluated in three steps: before the project running and patient selection, during the execution of the trial, and after sampling termination. The number of patients referred to Shariati, Imam Reza, and Yasuj hospitals who were in elapsed/remission status was one case per month, which was the suitable interval for cell production and other patients’ follow-up. Data access in all patients were well access except in P01, where died. (Supplementary file2). NK cell production feasibility was proved by NK cell co-culture results and NK cell immunophenotyping.

### Possible clinical efficacy

All 10 patients received all 2 planned PB-NK-cell infusions; One patient (p01) was not assessable because of COVID-19 infection. Nine patients were evaluated for response assessment after PB-NK cell infusion (Table 3). Of 9 evaluable patients, 6 (66.6%) showed stable disease (SD) and 3 (33.3%) presented progressive disease (PD). In 6 SD patients, the blast percentage within 3 months remained stable after the first and second courses of therapy with PB-NK cells (Table 3). Of 6 SD patients, 2 (p08 and p09) remained alive in SD and 3 patients (p04, p05 and p07) converted to PD at 9 months after infusion of NK cells, and 1 (p03) was not evaluable due to follow-up loss. Patients were followed until death, and all of them (except P03) had died at last follow up due to progressive disease (Table 3).

**Table 3:** Treatment schedule and outcomes

Patients	Cohort	1st NK infusion dose (×10^6^/kg)	2nd NK infusion dose (×10^6^/kg)	No. Of doses	% Blasts in BM 90 days after infusion	Disease status at day 90	Last follow up (One year Follow-Up^a^)
P01	1	2	5	2	*	Died (due to COVID)	Dead
P02	1	2	5	2	60%	Progressive disease	Dead
P03	1	2	5	2	38%	Stable disease	Follow-up loss
P04	1	2	5	2	15%	Stable disease	Dead
P05	1	2	5	2	22%	Stable disease	Dead
P06	2	5	10	2	37%	Progressive disease	Dead
P07	2	5	10	2	18%	Stable disease	Dead
P08	2	5	10	2	5%	Stable disease	Dead
P09	2	5	10	2	22%	Stable disease	Dead
P10	2	5	10	2	34%	Progressive disease	Dead

* Data is not accessible

^a^ All patients (except P03) had died at last follow up due to progressive disease

### The laboratory finding

The WBC count of all patients decreased immediately after conditioning regimen administration. Liver enzymes such as SGOT, SGPT, creatinine, and ALP were in the normal range from hospitalization until discharge, and our intervention had no significant impact on these values. Electrolyte non-difference was a marker of normal homeostasis due to the intervention. Descriptive results are shown in Table 4 for each patient.

**Table 4:** Descriptive data of laboratory parameters of patients after 2nd cell infusion

Patients	P01		P02		P03		P04		P05		P06		P07		P08		P09		P10	
	Before	After	Before	After	Before	After	Before	After	Before	After	Before	After	Before	After	Before	After	Before	After	Before	After
SGOT	25	17	15	12	15	16	10	8	22	21	11	13	17	19	16	15	13	16	18	22
SGPT	93	34	13	9	11	13	22	18	28	18	14	12	20	23	21	25	24	26	23	25
Cr	1.1	0.7	NA	NA	0.7	0.7	1	1.1	0.9	1	0.9	1.1	0.68	0.91	0.72	0.86	0.87	0.96	0.76	1.02
ALP	150	86	132	122	405	378	220	300	160	158	52	59	62	68	49	58	70	76	79	88
Na	139	134	132	135	132	133	135	134	139	136	131	135	139	146	132	149	148	159	153	156
K	4	4.7	3.8	4	4	3.8	4	3.8	3.8	3.7	3.8	4.1	4.2	4.6	4.3	4.9	3.9	4.3	3.5	4.2
WBC	5050	7200	800	600	2700	300	700	400	1800	870	1050	1456	1150	1790	700	1210	900	1340	1700	2620

ALP: alkaline phosphatase, aspartate aminotransferase (AST, SGOT), alanine aminotransferase (ALT, SGPT), creatinine (Cr), WBC: white blood cell, Na: sodium, K: potassium

## Discussion

Acute myeloid leukemia is one of the most lethal hematologic malignancies, for which many advanced therapies are used worldwide. Employing natural killer cells as the main arm of innate immunity can be effective as a complementary treatment. Allogeneic off-the-shelf cell therapy using natural killer cells can change the patients’ fate, and its developmental studies are essential for commercialization after safety, feasibility and efficacy study [27].

In this project, we expanded PB-NK cells by stimulation with irradiated autologous feeder cells to produce expanded and functional PB-NK cells [22]. The functionality and viability of all products were checked using experimental methods such as CD107a and IFN-g measurement.

This phase I study using allogeneic NK cells allowed us to draw some clinical insights for patients with relapsed/refractory AML. The original hypothesis for this trial was that, given the previous successful therapy of leukemia by NK cells from alloreactive haploidentical KIR ligand-mismatched cells, administration of MG4101 would be safe and display improved clinical benefit over other treatments [14, 23, 28, 29].

Organ function characterized by biochemical assays in patients’ serum. The serum levels of SGOT, SGPT, Cr, and ALP were in the normal range in all patients except one of those patients (P01) who died due to COVID-19; hence, she was not evaluated. Normal liver function showed that there is no side effect of conditioning regimen and cell infusion on normal homeostasis [30, 31].

These results agree with those of previous studies of individuals treated with adoptive allogenic NK cell therapy [11, 18, 32–34]. Our differences in the current study are the use of random healthy unrelated donor NK cells for two doses in relapsed/refractory AML, conditioning regimen without total lymphoid irradiation (TLI) and to avoid Tregs stimulation subcutaneous IL2 administration was omitted. Allogeneic NK cells derived from potential entirely random donors, using it for treatment could result in GVHD; however, these results support the safety and feasibility of this product in relapsed/refractory AML patients. Consistent with our finding [14–17, 23, 29], GVHD, cytokine release syndrome, and neurotoxicity were not observed. Dose escalation was well tolerated, and the target cell dose was 10 × 10^6^/ kg. For allogenic NK cell in vivo persistence reasons, our protocols included immunosuppressive regimens that would have not prohibited the outgrowth of allogenic NK cells derived from random unrelated donors. However, the in vivo persistence of NK cells can be enhanced through several approaches. These results agree with those of previous studies of individuals treated with adoptive NK cell therapy. Miller et al. [16] treated AML patients with IL2-activated CD3-depleted haploidentical NK cells after Flu/Cy –induced immunosuppression and detected notable in vivo proliferation and persistency of infused NK cells. However, we could not display in vivo expansion or prolonged persistence, possibly because of the absence of IL-2 administration and subsequent rejection by autologous lymphocytes.

Some groups are exploring allogenic NK cell expansion using platforms with and without feeder [23, 29]. Yang et al. [23] treated 20 patients with allogenic NK cells derived from random healthy donor cells. Of 17 assessable patients, eight (47.1%) displayed stable disease and nine (52.9%) displayed progressive disease. Adoptive NK cells were well tolerated, with only transient adverse events observed in all cases. However, these findings require larger-scale studies to confirm allogenic NK cell infusion efficacy in relapsed/refractory AML. Patients with relapsed/refractory AML might benefit from adoptive NK cell infusions to restore dysfunctional autologous NK cell response, at least in part, GVL. Haploidentical NK cells have been previously assessed in phase I and II in patients with AML both in the transplant and non-transplant settings [34–36]. NK-cell alloreactivity based on KIR mismatching using the receptor–ligand model in patients with AML undergoing haploidentical HSCT displayed improved survival rates [37, 38]. Adoptive transfer of allogenic NK cells along with conditioning regimens promotes expansion of the haploidentical NK cells with administration of IL-2-induced CR in relapsed and refractory AML patients [14, 16, 17]. In addition, several investigations have revealed that increased frequencies of Tregs can be directly associated with tumor progression [39]. In particular, Tregs can inhibit NK activation through TGFβ secretion. In considering allogenic NK cell infusion, it is necessary to consider the use of a conditioning regimen to enhance NK cell expansion and approaches to promote NK function post-infusion, such as depletion of T regulatory cells or the use of cytokines [16]. In this study, such an approach was used as PB-NK cells were transferred to a deeply immunosuppressive milieu already compromised by the conditioning regimen.

For patients with AML that are not candidates for HSCT and are chemo-refractor, treatment options are limited. Because PB-NK cells mediate strong GVL, their transfer may be beneficial for patients with relapsed/refractory AML. We conducted a phase 1 trial that demonstrated that PB-NK cell delivery was safe and feasible. No DLT was observed in relapsed/refractory AML patients who received 10 infusions of PB-NK cells. Some cases that developed infusion-related toxicity were responsive to supportive care.

We expected that the transfer of PB-NK cells would result in at least stable disease in relapsed/refractory AML due to leukemia blast reduction by transferred PB-NK cells. Thus, the patients’ leukemia blast status was monitored before and after therapy. After allogenic NK cell infusion, in some patients, no significant change in the percentage of leukemia blasts was detected. The most important goal of this project was the feasibility and safety assessment of NK cell production and infusion after conditioning regimen in patients with AML. Due to the CONSORT checklist, our study evaluates well in case of feasibility parameters.

This trial was not designed to characterize PB-NK cells after administration. This limitation will be evaluated by a complete analysis of donor NK-cell persistence and blood cytokines on infused PB-NK cells in a planned phase II trial. Chimerism levels of NK cells at 7 or 14 days after administration have been correlated with CR after IL-2-activated haploidentical NK cells [40].

In summary, this trial supports the safety and feasibility of expanded PB-NK cells for effective “off-the-shelf” immunotherapy, while their innate alloreactivity can be safely harnessed to potentiate allogeneic cell therapy. This capability, together with the apparently minimal HLA-matching requirements between the donor and recipient of NK cells, may pave the way for a truly off-the-shelf product that could increase treatment accessibility for more patients. The most important limitation in this study was the small sample size, and it was about the aim of study in case of NK cell infusion safety and feasibility evaluation in dose escalation setting. Eventually, since 10 × 10^6^ cells were well tolerated in all patients, we can design a randomized clinical trial for efficacy assessment.

### Electronic supplementary material

Below is the link to the electronic supplementary material.

Supplementary Material 1

Supplementary Material 2

## Data Availability

All data generated or analyzed during this study have been included in this published article.
