# Supplementary material for: Phase I non-randomized clinical trial of allogeneic natural killer cells infusion in acute myeloid leukemia patients
Source: BMC Cancer. 2023 Nov 10;23:1090. doi: 10.1186/s12885-023-11610-x (PMC10636850; doi:10.1186/s12885-023-11610-x)
Supplement: Supplementary file 1 — Supplementary Material 1 [file 12885_2023_11610_MOESM1_ESM.doc]

**The Feasibility and Safety study of allogeneic NK cell therapy in patients with refractory/relapsed AML**

Project subject:

Laision Researcher:

Chief Medicinal Doctrate:

Patient's File Code:

Patient's Study Code:

Date/Time of intervention

# Group Code

**Part 1: Sample Size evaluation (Filled by Ward's Nurse Supervisor)**

- Number of eligible admitted patients according to inclusion Criteria ……
- Number of discharge/death patients eligible to trial according to inclusion criteria……

Part 2: Data Access (insert + for each option that is feasible)

| Pateints Codes | Demographic information | Hardcopy of patient file | Local access to HIS of hospital | Remote access to HIS of hospital | Full access to patients information after discharge |
| --- | --- | --- | --- | --- | --- |
|  |  |  |  |  |  |
|  |  |  |  |  |  |
|  |  |  |  |  |  |
|  |  |  |  |  |  |
|  |  |  |  |  |  |

HIS: health information system

Part 3. Cause of Death (insert + for each option that is feasible)

| Pateints Codes | No information about death cause | General information of death cause | Professional assessment of death cause |
| --- | --- | --- | --- |
|  |  |  |  |
|  |  |  |  |
|  |  |  |  |
|  |  |  |  |
|  |  |  |  |

**Prinsiple Investigator**

**sign**

**Prinsiple Medical Doctroate**

**sign**
