# Supplementary material for: Phase I non-randomized clinical trial of allogeneic natural killer cells infusion in acute myeloid leukemia patients
Source: BMC Cancer. 2023 Nov 10;23:1090. doi: 10.1186/s12885-023-11610-x (PMC10636850; doi:10.1186/s12885-023-11610-x)
Supplement: Supplementary file 2 — Supplementary Material 2 [file 12885_2023_11610_MOESM2_ESM.doc]

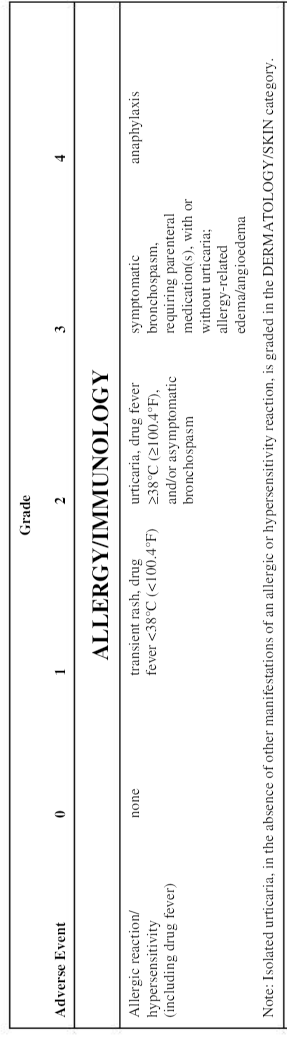


| Rash maculopapular | Pain in extremity | Bone pain | Sinus tachycardia | Encephalitis infection | Vomiting | Headache | Nausea | Chills |  |
| --- | --- | --- | --- | --- | --- | --- | --- | --- | --- |
|  |  |  |  |  |  |  |  | * | P01 |
|  |  |  |  |  |  |  |  | +8 | Date: count |
|  |  |  |  |  |  |  |  |  | P02 |
|  |  |  |  |  |  |  |  |  | Date: count |
|  |  |  |  |  |  |  |  |  | P03 |
|  |  |  |  |  |  |  |  |  | Date: count |
|  |  |  |  |  |  | * |  |  | P04 |
|  |  |  |  |  |  | +9 |  |  | Date: count |
|  |  |  |  |  | * |  |  |  | P05 |
|  |  |  |  |  | +5 |  |  |  | Date: count |
|  |  |  |  |  |  |  |  |  | P06 |
|  |  |  |  |  |  |  |  |  | Date: count |
|  |  |  |  |  |  |  |  |  | P07 |
|  |  |  |  |  |  |  |  |  | Date: count |
|  |  | * |  |  |  |  |  |  | P08 |
|  |  | +10 |  |  |  |  |  |  | Date: count |
|  |  |  |  |  |  |  |  |  | P09 |
|  |  |  |  |  |  |  |  |  | Date: count |
|  |  |  |  |  |  |  |  |  | P10 |
|  |  |  |  |  |  |  |  |  | Date: count |
|  |  | 1 |  |  | 1 | 1 |  | 1 | Sum |

| Laboratory tests availability | Cause of death file | Data access | Patient accessibility | Step of study |  |
| --- | --- | --- | --- | --- | --- |
| 8/10 | 5 | 5 | 5 | Before study | P01 |
| 8/10 | 5 | 5 | 5 | Follow-up |
| 8/10 | - | 5 | 4 | Before study | P02 |
| 8/10 | - | 3 | 4 | Follow-up |
| 8/10 | - | 5 | 4 | Before study | P03 |
| 8/10 | - | 3 | 4 | Follow-up |
| 8/10 | - | 2 | 5 | Before study | P04 |
| 8/10 | - | 3 | 4 | Follow-up |
| 8/10 | - | 4 | 4 | Before study | P05 |
| 8/10 | - | 4 | 4 | Follow-up |
| 8/10 | - | 5 | 5 | Before study | P06 |
| 8/10 | - | 4 | 5 | Follow-up |
| 8/10 | - | 4 | 4 | Before study | P07 |
| 8/10 | - | 5 | 5 | Follow-up |
| 8/10 | - | 4 | 5 | Before study | P08 |
| 8/10 | - | 5 | 5 | Follow-up |
| 8/10 | - | 4 | 4 | Before study | P09 |
| 8/10 | - | 5 | 5 | Follow-up |
| 8/10 | - | 4 | 5 | Before study | P10 |
| 8/10 | - | 5 | 3 | Follow-up |

Score1-5: 1 (non accecs), 2 (hard access), 3 (time consuming), 4 (good acess), 5 (full access)
